# Young age at first pregnancy does protect against early onset breast cancer in *BRCA1* and *BRCA2* mutation carriers

**DOI:** 10.1007/s10549-017-4557-1

**Published:** 2017-11-07

**Authors:** DG Evans, EF Harkness, S. Howel, ER Woodward, A. Howell, F. Lalloo

**Affiliations:** 1 Manchester Centre for Genomic Medicine, Manchester Academic Health Sciences Centre (MAHSC), Institute of Human Development, St Mary’s Hospital, University of Manchester, Manchester, M13 9WL UK; 2 Prevent Breast Cancer Centre, University Hospital of South Manchester NHS Trust, Wythenshawe, Manchester M23 9LT UK; 3 Manchester Centre for Genomic Medicine, St Mary’s Hospital, Central Manchester University Hospitals NHS Foundation Trust, Manchester, M13 9WL UK; 4 Manchester Breast Centre, The University of Manchester, Manchester, M20 4BX UK; 5 Division of Informatics, Imaging and Data Sciences, Faculty of Biology, Medicine and Health, Manchester Academic Health Science Centre, University of Manchester, Stopford Building, Oxford Road, Manchester, M13 9PT UK; 6 Department of Medical Oncology, The Christie, Manchester, M20 4BX UK

**Keywords:** BRCA1, BRCA2, Breast cancer, Age at first pregnancy

## Abstract

**Purpose:**

Previous research assessing the impact of pregnancy and age at first pregnancy on breast cancer risk in *BRCA1* and *BRCA2* mutation carriers has produced conflicting results, with some studies showing an increased risk following early first pregnancy in contrast to the reduced risk in the general population of women. The present study addresses these inconsistencies.

**Methods:**

Female *BRCA1* and *BRCA2* carriers from North West England were assessed for breast cancer incidence prior to 50 years of age comparing those with an early first full-term pregnancy (< 21 years) to those without a full-term pregnancy. Breast cancer incidence per decade from 20 years and Kaplan–Meier analyses were performed.

**Results:**

2424 female mutation carriers (1278 *BRCA1*; 1146 *BRCA2*) developed 990 breast cancers under the age of 50 years. Women who had their first term pregnancy prior to age 21 (*n* = 441) had a lower cancer incidence especially between age 30–39 years. Kaplan–Meier analysis showed an odds ratio of 0.78 for *BRCA1* (*p* = 0.005) and 0.73 for *BRCA2* (*p* = 0.002).

**Conclusions:**

The present study demonstrates a clear protective effect of early first pregnancy on breast cancer risk in both *BRCA1* and *BRCA2* mutation carriers.

## Introduction (2047)

Older age at first pregnancy is a strong predictor for increased risk of later onset breast cancer both in the general population [1] and in women with a family history of breast cancer [2]. However, published evidence for *BRCA1* and *BRCA2* pathogenic mutation carriers (with a very high lifetime risk of breast cancer) is conflicting [3–8]. Taking studies chronologically from 1999, an initial study showed no effect of age at first pregnancy but an increase in risk of breast cancer by age 40 in women who were parous compared to nulliparous [3]. Cullinane et al. showed some protective effect of multiple pregnancies in *BRCA1* mutation carriers but an increase in breast cancer risk in *BRCA2* carriers [4]. A further study showed an increased risk in *BRCA1* carriers with early compared to late first pregnancy [5] (although, in *BRCA2,* early first pregnancy was protective), but no overall protective effect of pregnancy for either gene. In 2007, a case control study demonstrated no effect of either pregnancy or age at first live birth in breast cancer risk for either gene [6]. Milne et al. demonstrated a protective effect of any pregnancy, but only for breast cancer diagnosed after age 40 and no effect of early age at first pregnancy for either gene [7]. More recently, in 2012, Lecarpentier et al. demonstrated protection from multiple pregnancies (> 2), but for *BRCA1*, this was confined to individuals with pathogenic mutations in the central portion of the gene [8]. There is, therefore, no consistent evidence for an effect of age at first pregnancy. Indeed, some studies demonstrate an increased risk of breast cancer with any pregnancy in mutation carriers in contrast to the general population [3–5]. In view of these inconsistencies, we have examined the effects of early age at first full-term pregnancy (FFTP) on breast cancer risk to age 50 years in the Manchester regional BRCA database.

## Methods

Testing for *BRCA1/2* mutations began in Manchester in 1996. A total of 4086 families have been tested for *BRCA1/2* pathogenic mutations by sequencing all exons of the *BRCA1* and *BRCA2* genes (since 2014 by next generation sequencing) and testing for whole exon deletions or duplications by multiple ligation dependent probe amplification (MLPA). Once a pathogenic variant is identified, predictive genetic testing for the familial mutation is offered to other affected and unaffected at risk relatives. Mutation carriers are identified by testing positive for the family pathogenic mutation, or by inference as an obligate carrier by segregation within a family. Family members have been tested in a total of 623 families with *BRCA1* pathogenic mutations (98 notified from other centres) and 584 *BRCA2* pathogenic mutations (103 notified from other centres). Age at first full-term pregnancy (FFTP) was calculated from date of delivery of first full-term child and date of birth of mother. Dates of first breast cancer were identified from pathology records or from the North West Cancer Intelligence service. Women were censored at date of breast cancer, risk reducing mastectomy, risk reducing oophorectomy (there is at least an effect in *BRCA2*), ovarian cancer, date of death, or date of last follow-up, whichever was earliest. Follow-up was taken from age 20 years to 50 years. Women who were nulliparous were treated as having no FFTP (group 0). Women with FFTP aged 20-and-under were treated as the study comparator group (group 1). For women with a FFTP after the age of 20 (21–46 years), follow-up from age 20 to date of FFTP was included in the nulliparous group (group 0), whilst follow-up after FFTP to censor date was included in group 2 for FFTP 21–29 years, group 3 for FFTP 30–39 years and group 4 if FFTP > 39. Therefore, all follow-up prior to FFTP in any women with a first pregnancy after the age of 20, was included in the nulliparous group. In order to correct for ascertainment bias, a second analysis was carried out to include only follow-up and breast cancers from date of family ascertainment to the Department of Genomic Medicine.

Assessment of breast cancers occurring aged < 50 years in untested female first degree relatives was undertaken to assess any potential testing bias.

A subgroup analysis was carried out in our previously reported 30-years and-under breast cancer study [9]. Women diagnosed with breast cancer under the age of 31 from 01/01/1980 to 31/12/1997 from North West England (population 4.1 million at time of study) were included. This study recruited from 1991 to 1998. All women consenting to the study were offered *BRCA1*, *BRCA2* and *TP53* testing.

Median age at first pregnancy as a control was taken from women entered onto the Manchester Regional BRCA family register database as mutation carriers or tested or untested first degree relatives.

Rates of breast cancer per 1000 women were calculated by age group (20–29, 30–39 and 40–49) at breast cancer diagnosis for each group (nulliparous, FFTP < 21, FFTP 21–29, FFTP 31–39, FFTP > 39). Cumulative incidence curves were compared using Kaplan–Meier analysis and the log rank test. The cumulative incidence rate at age 40 and 50 is reported and odds ratios calculated as the ratio of the cumulative incidence rate for group 1 compared to group 0. Analysis was conducted using Stata version 14.

Ethics approval for the study was through the North Manchester Research (08/H1006/77) and University of Manchester (08229) ethics committees.

## Results

### Main analysis on BRCA carriers

**Fig. 1:**
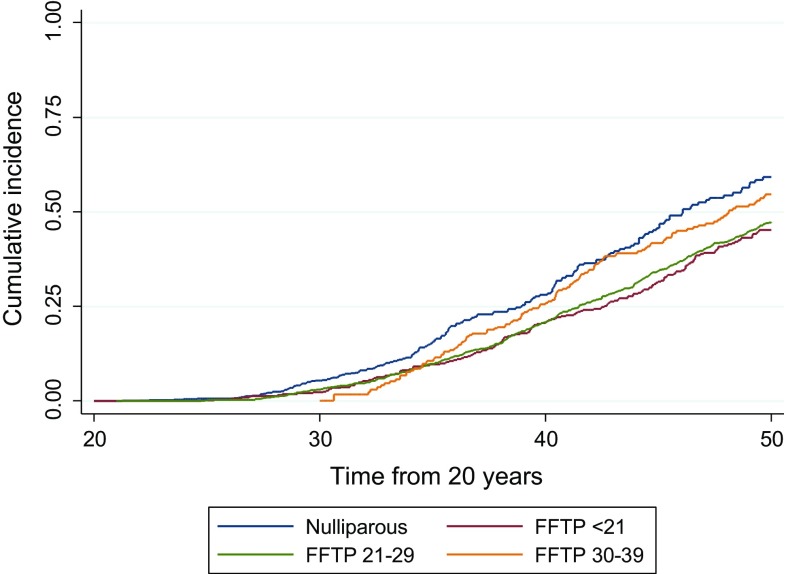
*BRCA1* and *BRCA2* combined showing cumulative onset of breast cancer between age 20–50 years in groups 0, 1, 2 and 3. Note group 3 had no follow-up prior to 30 years of age thus the curve only starts at this age

**Fig. 2:**
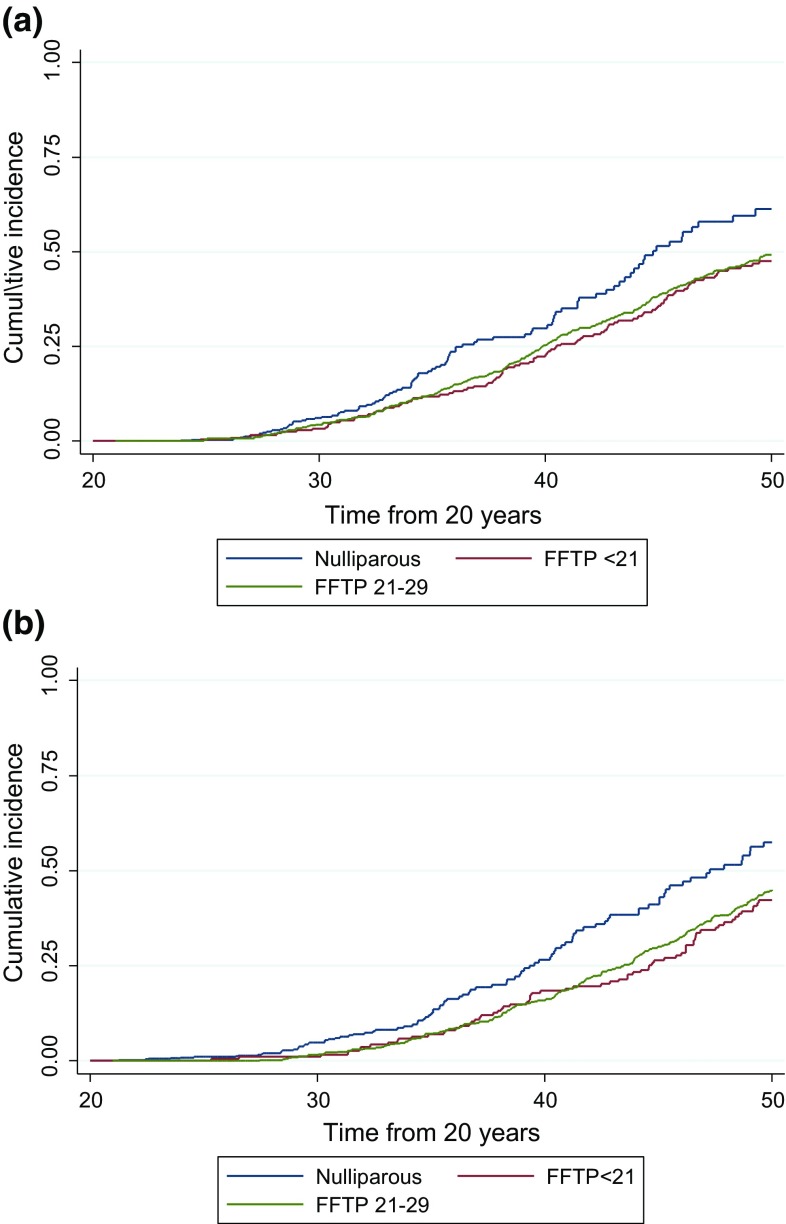
*BRCA1* and *BRCA2* female mutation carriers showing cumulative onset of breast cancer to age 50 years in groups 0, 1 and 2. **a** BRCA1, and **b** BRCA2

**Fig. 3:**
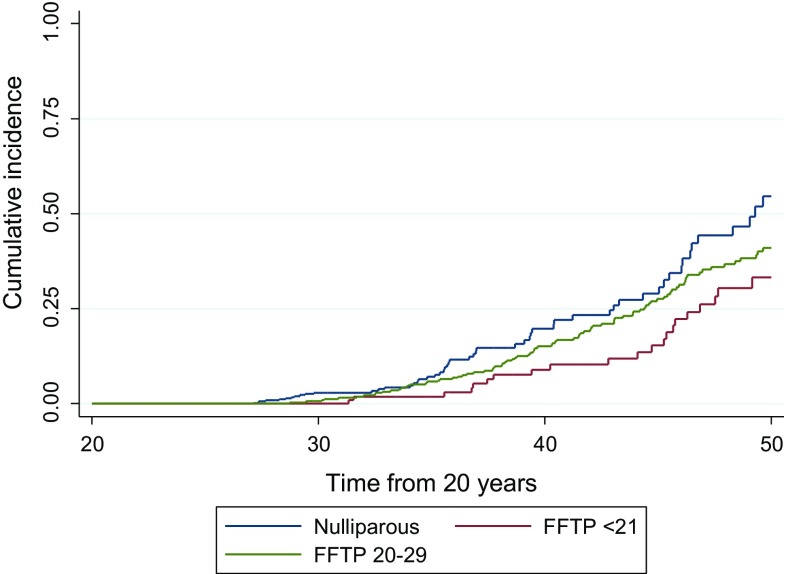
*BRCA1* and *BRCA2* combined showing cumulative onset of breast cancer to age 50 years in groups 0, 1 and 2 for the prospective analysis

There were 441 females with pathogenic mutations (247-*BRCA1*; 194-*BRCA2*) with age at FFTP of < 21 years. A total of 448 female carriers had not had a full-term pregnancy at last follow-up (230-*BRCA1*; 218-*BRCA2*). Follow-up in 1535 women with FFTP aged 21–29 (group 2; *n* = 1209), 30–39 (group 3; *n* = 334) and 40–46 (group 4; *n* = 12) was available (801-*BRCA1*; 734-*BRCA2*) resulting in 2424 female carriers in total. These 1535 women in groups 2–4 contributed 10,302 years of follow-up between age 20 and the date of their first birth and developed 20 breast cancers before their first birth or within 1 month thereof. These data were included in the nulliparous control group (group 0) in Table 1. One hundred and sixty-six women developed breast cancer in the study group (FFTP aged < 21; group 1) to age 50 years and 202 in group 0. Age at menarche was not significantly different between group 0 (mean 12.79) and group 1 (mean 13.04). Breast cancer rates were higher in those that had FFTP aged 30–39 and in the nulliparous control group 0 (Table 1; Figs. 1, 2). These differences continued to be seen if women were left censored at date of family ascertainment (Table 2; Fig. 3). Kaplan–Meier analysis shows clear separation of the curves with a risk of 59.3% by age 50 in group 0 and 45.2% in group 1 Log Rank (Mantel-Cox) *p* < 0.001 odds ratio for group 1 compared to group 0 = 0.76. Results remained significant for both *BRCA1* OR 0.78 (*p* = 0.005) and *BRCA2* OR 0.73 (*p* = 0.002). At age 40, corresponding values are 28.1% for group 0 and 20.6% for group 1, *p* = 0.004.

**Table 1:** Breast cancer rates in female *BRCA1* and *BRCA2* mutation carriers by groups 0–4

a: Censored to age 50 (*BRCA1* and *BRCA2*)						
Age BC	Follow-up from age 20		Follow-up from FFTP			Total (grp 0 + 2 + 3 + 4)
	FFTP < 21	Nulliparous^+^	21–29	30–39	40 +	
	Grp 1	Grp 0	Grp 2	Grp 3	Grp 4	
20–49^a^						
Years	10,295.03	17,943.48	22,909.00	3170.26	65.33	44,088.06
Rate	16.12	11.26	21.52	40.06	30.62	18.69
BCs	166	202	493	127	2	824
20–29						
Years	4343.46	13,378.45	6044.12			19,422.57
Rate	2.30	3.96	5.13			4.32
BCs	10	53	31			84
30–39						
Years	3666.28	3501.74	10,437.12	1844.25		15,783.11
Rate	20.18	25.13	19.64	32.53		22.37
BCs	74	88	205	60		353
40–49						
Years	2285.29	1063.29	6427.75	1326.01	65.33	8882.38
Rate	35.88	57.37	39.98	50.53	30.62	43.57
BCs	82	61	257	67	2	387

b: Censored to age 50 (BRCA1)						
Age BC	< 21 FFTP	Nulliparous	Follow-up from FFTP			Total (grp 0 + 2 + 3 + 4)
			21–29	30–39	40 +	
	Grp 1	Grp 0	Grp 2	Grp 3	Grp 4	
20–49						
Years	5585.42	9022.48	11,581.57	1363.82	23.25	21,991.12
Rate	17.37	11.53	23.23	44.73	43.01	19.78
BCs	97	104	269	61	1	435
20–29						
Years	2427.15	6917.98	3141.88			10,059.87
Rate	3.30	4.19	7.32			5.17
BCs	8	29	23			52
30–39						
Years	2000.07	1659.86	5351.96	839.47		7851.28
Rate	21.50	28.32	23.73	42.88		26.75
BCs	43	47	127	36		210
40–49						
Years	1158.20	444.64	3087.73	524.36	23.25	4079.97
Rate	39.72	62.97	38.54	47.68	43.01	42.40
BCs	46	28	119	25	1	173

c: Censored to age 50 (BRCA2)						
Age BC	< 21 FFTP	Nulliparous	Follow-up from FFTP			Total (grp 0 + 02 + 03 + 04)
			21–29	30–39	40 +	
	Grp 1	Grp 0	Grp 02	Grp 03	Grp 04	
20–49						
Years	4709.61	8921.00	11,327.42	1806.43	42.08	22,096.94
Rate	14.65	10.99	19.78	36.54	23.77	17.60
BCs	69	98	224	66	1	389
20–29						
Years	1916.31	6460.46	2902.24			9362.71
Rate	1.04	3.71	2.76			3.42
BCs	2	24	8			32
30–39						
Years	1666.21	1841.88	5085.16	1004.78		7931.83
Rate	18.61	22.26	15.34	23.89		18.03
BCs	31	41	78	24		143
40–49						
Years	1127.09	618.65	3340.02	801.65	42.08	4802.40
Rate	31.94	53.34	41.32	52.39	23.77	44.56
BCs	36	33	138	42	1	214

^+^ Includes pre FFTP follow-up

^a^Note rates are lower for group 0 in total follow-up, as most follow-up was in the 20–29 age group. Follow-up prior to FFTP is included in group 0

**Table 2:** Breast cancer rates in prospective series for groups 0–4

Age BC	< 21 FFTP	Nulliparous	Follow-up from FFTP			Total (grp 0 + 2 + 3 + 4)
			21–29	30–39	40 +	
	Grp 1	Grp 0	Grp 2	Grp 3	Grp 4	
Censored to age 50 (*BRCA1* and *BRCA2*)—prospective analysis						
20–49^a^						
Years	2509.65	7350.09	5547.07	1229.42	4.16	14,130.74
Rate	8.37	7.34	15.50	24.40	240.30	12.10
BCs	21	54	86	30	1	171
20–29						
Years	1138.48	5306.68	1560.09			6866.77
Rate	0.00	2.08	1.28			1.89
BCs	0	11	2			13
30–39						
Years	906.24	1627.97	2722.95	769.48		5120.40
Rate	8.83	14.74	14.69	15.59		14.84
BCs	8	24	40	12		76
40–49						
Years	464.93	415.43	1264.03	459.94	4.16	2143.57
Rate	27.96	45.74	34.81	39.14	240.30	38.25
BCs	13	19	44	18	1	82

^a^Note rates are lower for group 0 as most follow-up was in the 20–29 age group

In prospective analysis, group 1 was significantly less likely to develop breast cancer with 33.2% risk by age 50 (95% CI 22.4–47.3) compared to 54.6% (95% CI 42.1–68.0) in group 0 (*p* = 0.005). group 2 was intermediate at 40.9% (95% CI 34.1–48.6) risk by age 50 years still significantly less than the nulliparous group (*p* = 0.01).

Breast cancer developed in 622 of 1535 *BRCA1/2* mutation carriers (40.5%) who had completed their first pregnancy between 21 and 47 years of age had developed breast cancer after their FFTP but before 50 years of age. Risks for these groups are presented in Tables 1 and 2.

### Untested female relatives

Eighty-three first degree relatives of mutation carriers who were affected with breast cancer had not undergone mutation testing, which included 55 women who had completed a pregnancy and 14 (20%) who had not (14 not known). This constituted 5/20 (25%) of those with breast cancer aged < 40 years. Amongst those unaffected, first degree relatives who had not undergone presymptomatic testing and were aged 40 years of age or older 106/543 (19.5%) were nulliparous. As such, only 20/541 (3.7%) breast cancers aged < 40 years were of unknown mutation status and 548/1639 (33.4%) of those unaffected female first degree relatives who were over 40 years of age had not undergone testing.

### 30-and-under study

In the 30-and-under study, blood was screened on 122 women (72.2%) out of a possible 169 who were alive during study recruitment (1991–1998). Twenty-seven women declined the study and in 20, the clinician responsible refused access to the woman. A total of 31/122 (25.4%) had mutations in *BRCA1* (*n* = 22) or *BRCA2* (*n* = 9). 16/31 (51.6%) had completed a FFTP before their breast cancer diagnosis with 15 either having their first pregnancy after breast cancer (*n* = 4) or remaining nulliparous (*n* = 11). Median age at breast cancer diagnosis was 28.9 years. From the Manchester BRCA database of women with years of birth from 1950 to 1977 (the same range as in the BRCA cases from 30-and-under), 58.9% (1486/2521) had completed their FFTP before their 29th birthday. Amongst 31 BRCA carriers, this would have given an expected number for those having completed an FFTP by 29 of 18.25, whereas slightly less than this (*n* = 16) had completed a FFTP by this time. This means there was a non-significant over representation of women who had not yet had a completed pregnancy by the time they were diagnosed with breast cancer, suggesting there was not a testing bias towards nulliparous women.

## Discussion

The present study demonstrates a clear protective effect of early first pregnancy on breast cancer risk in women with pathogenic *BRCA1* or *BRCA2* mutations. Nulliparity results in an approximately 30% higher likelihood of developing breast cancer by 50 years of age than if a woman had completed a pregnancy by age 21 years. The present study also shows that the effect is sustained in unbiased prospective analysis. It is possible that previous studies have been subject to ascertainment bias. Women with early onset breast cancer who have children may have been more likely to opt for BRCA testing to help ascertain risks for their children. This bias may decrease with BRCA testing now being driven by the need to alter treatment to platinum based chemotherapy or PARPi treatments [10, 11]. BRCA testing may also alter decision making regarding surgical treatment including contralateral mastectomy [12–14].

Previous analysis from our group 10 years ago on 789 mutation carriers, demonstrated a protective effect of pregnancy in both *BRCA1* and *BRCA2* pathogenic mutation carriers and with young age at first pregnancy only in *BRCA2* mutation carriers [15]. However, that analysis did not show any effect prior to 40 years of age. The present study, with at least 3 times the number of carriers, shows the protective effect on breast cancer between 20 and 50 years of age, of early full- term first pregnancy with odds ratios of 0.77 and 0.73 for *BRCA1* and *BRCA2* mutation carriers, respectively, and in prospective analysis, an even better odds ratio for both combined at 0.61. Another recent study of 197 BRCA mutation carriers has also shown an effect with mean age at breast cancer of 36.2 years in nulliparous carriers compared to 40.9 years (*p* = 0.001) in parous carriers [16].

Whilst informing women about the potential benefits of early first pregnancy may be difficult, particularly if they are already over 30 years of age, some women may wish to complete their family earlier if they know this could reduce their risk. Any pregnancy in a BRCA carrier over 30 years of age will have the a greater potential (around 1–2%) of a pregnancy associated breast cancer, which is likely to be detected at a later stage as mammography and MRI are not possible during, and for some time after, pregnancy and/or breast feeding. Even though it is unlikely to change many women’s minds, (even if they are able to plan it) some women in stable relationships who may otherwise have delayed pregnancy for career aspirations, may decide on early pregnancy to avoid the later risk of pregnancy associated breast cancer and to reduce their overall breast cancer risk.

There are some limitations to the current study. Not all eligible women chose to have testing. This may result in a testing bias. However, if anything, the proportion of nulliparous women was higher at 25% of those diagnosed with breast cancer at < 40 years of age, suggesting that if any testing bias existed, it was towards not testing young onset breast cancers in nulliparous women. Overall, there were only 20 untested relatives with breast cancer < 40 years of age compared to 235 in group 0 and group 1 in the study who were known to be mutation carriers. The breast cancer rates and penetrance to age 40 and 50 years are likely to be overestimates since some first degree relatives were not tested, the majority of which were unaffected. These rates should not be quoted to unaffected mutation carriers. However, results of the prospective analysis with risk of 33% by age 50 in women with an early FFTP compared to 55% in nulliparous women are likely more accurate although confidence intervals are larger due to smaller numbers.

In conclusion, the current study of nearly 2500 female BRCA pathogenic mutation carriers shows a clear protective effect of early FFTP, which adds to the weight of evidence that risk factors are very similar in BRCA mutation carriers to those in the general population. Women who undergo testing in the late teenage years and early twenties should be informed of this.
